# Probing signal amplification by reversible exchange using an NMR flow system

**DOI:** 10.1002/mrc.4073

**Published:** 2014-05-06

**Authors:** Ryan E Mewis, Kevin D Atkinson, Michael J Cowley, Simon B Duckett, Gary G R Green, Richard A Green, Louise A R Highton, David Kilgour, Lyrelle S Lloyd, Joost A B Lohman, David C Williamson

**Affiliations:** a Centre for Hyperpolarisation in Magnetic Resonance, University of YorkHeslington, York, YO10 5DD, UK; b Bruker BioSpin GmbHSilberstreifen 4, Rheinstetten, 76287, Germany; c Bruker UK LimitedBanner Lane, Coventry, CV4 9GH, UK

**Keywords:** NMR, parahydrogen, SABRE, hyperpolarization

## Abstract

Hyperpolarization methods are used in NMR to overcome its inherent sensitivity problem. Herein, the biologically relevant target nicotinamide is polarized by the hyperpolarization technique signal amplification by reversible exchange. We illustrate how the polarization transfer field, and the concentrations of *para*hydrogen, the polarization-transfer-catalyst and substrate can be used to maximize signal amplification by reversible exchange effectiveness by reference to the first-order spin system of this target. The catalyst is shown to be crucial in this process, first by facilitating the transfer of hyperpolarization from *para*hydrogen to nicotinamide and then by depleting the resulting polarized states through further interaction. The 15 longitudinal one, two, three and four spin order terms produced are rigorously identified and quantified using an automated flow apparatus in conjunction with NMR pulse sequences based on the only parahydrogen spectroscopy protocol. The rates of build-up of these terms were shown to follow the order four∼three > two > single spin; this order parallels their rates of relaxation. The result of these competing effects is that the less-efficiently formed single-spin order terms dominate at the point of measurement with the two-spin terms having amplitudes that are an order of magnitude lower. We also complete further measurements to demonstrate that ^13^C NMR spectra can be readily collected where the long-lived quaternary ^13^C signals appear with significant intensity. These are improved upon by using INEPT. In summary, we dissect the complexity of this method, highlighting its benefits to the NMR community and its applicability for high-sensitivity magnetic resonance imaging detection in the future. © 2014 The Authors. *Magnetic Resonance in Chemistry* by John Wiley & Sons, Ltd.

## Introduction

Nuclear magnetic resonance spectroscopy is one of the most powerful techniques available to the chemist for examining molecular systems.^1^ Few other methods offer such an informative insight into events that proceed at a molecular level where precise information about structures, reaction pathways and dynamic behaviour flows from one observation method.^2^ Inherent low sensitivity reflects a serious drawback of NMR that stems from the fact that the detected signal strength relates to the population difference that is created between nuclear spin states because of the Zeeman effect and applies even in the high magnetic fields of the most advanced and costly NMR spectrometers. Several approaches have been discovered and are available to enhance the detected NMR signal strength. These exploit different physical properties, but all of them create non-equilibrium spin-state population differences between connected magnetic energy levels at the point of observation. Such methods include dynamic nuclear polarization,^3^ a process that utilizes the NOE^4^ to transfer magnetic encoding from highly polarized electrons at low temperature to appropriate nuclei. This process requires the cooling to around 1.3 K of the material to be probed together with a radical, whereupon microwave irradiation drives the polarization transfer step.^5^ Once sufficient polarization is transferred, the sample is thawed and moved to the spectrometer for interrogation by NMR. This very successful method has generated nuclear polarizations of 37% for ^13^C and 7.8% for ^15^N in labelled urea.^6^ Another technique uses laser irradiation to transfer polarization from rubidium vapour to noble gas nuclei.^7^ The ^3^He and ^129^Xe hyperpolarization created in this way has been used in magnetic resonance imaging (MRI) to enable high-sensitivity monitoring^7^ of pulmonary systems. Developments have now established that polarized xenon can be dissolved in a suitable cage for work in blood where, while it is diluted, the magnetization lifetime is extended.^8^

In this paper, we report our further developments on the transfer of non-equilibrium nuclear spin order, commonly referred to as polarization transfer or hyperpolarization transfer, from *para*hydrogen to a substrate of interest. While *para*hydrogen itself has no net spin angular momentum and is thus NMR silent, when it is used as a reagent in a reaction, many products can be formed that possess non-equilibrium nuclear spin distributions.^9^,^10^ This is readily understood if the NMR-invisible *αβ–βα* singlet spin state associated with molecular *para*hydrogen connects with visible *βα* and *αβ* Zeeman states in the product molecule resulting in hyperpolarized ^1^H-NMR signals.^11^ Transfer of spin order in this way in the high field of the magnet was first predicted by Bowers and Weitekamp.^12^ The effect was demonstrated by them^13^ and named *para*hydrogen and synthesis allow dramatically enhanced nuclear alignment (PASADENA). Eisenschmid *et al*.^14^ also reported their observations of similar, enhanced antiphase signal effects. The related effect, when the hydrogenation reaction takes place in the low field outside of the magnet prior to insertion into the magnet for measurement, was also discovered^15^ and was named adiabatic longitudinal transport after dissociation engenders net alignment (ALTADENA). These effects, for convenience, have been and are commonly and generically referred to as *para*hydrogen-induced polarization, or PHIP. PHIP that is created in this way has been shown to enable the study of reaction intermediates that would exist in such low concentrations as to prevent their detection by NMR spectroscopy.^16^–^19^ Nevertheless, using this approach, the reactants ultimately must be able to accept H_2_ and incorporate it into the product molecules and hence must be both unsaturated and highly reactive. This requirement generally limits the range of organic compounds that can be studied by this approach. However, polarization transfer to heteronuclei in these types of systems with AB, AX, AA′X, ABX, AMXY and AA′XX′Y nuclear spin topologies has been studied.^20^,^21^ The effects of variable magnetic fields on PHIP in multispin systems have also been investigated.^22^ Increasingly, in more recent years, applications of PHIP in the MRI field have been investigated and pursued.^23^–^28^ A number of *para*hydrogen polarizers for use in PHIP experiments have been described.^29^–^31^

Recent reviews^32^,^33^ illustrate how *para*hydrogen has been used in a wide variety of situations. These include a recent development where it hyperpolarizes a growing range of organic substrates through the establishment of a simple magnetic interaction while both are bound to a metal centre.^34^ This process requires both the substrate and *para*hydrogen to exchange freely in solution with their ligated forms in the complex in order to build up a concentration of hyperpolarized product. This has been termed signal amplification by reversible exchange (SABRE).^35^

Our previous reports on SABRE experiments have presented data that was acquired using a shake/dissolve method.^34^–^36^ This approach typically achieves polarization transfer in a sealed NMR tube through chemical exchange in an approximately defined magnetic field over a period of between 10 and 20 s. The shaking process provides a route to the rapid formation of the dihydride complex associated with polarization transfer. The highest effective concentration of *para*hydrogen in solution is simultaneously retained throughout the transfer step by facilitating rapid gas equilibration with the headspace. After shaking in this way, the sample is quickly transferred into the magnet of the NMR spectrometer for data acquisition. One of the underlying problems associated with such a simple approach is a lack of reproducibility in the absolute value of the NMR response. Experimentally, the extent of this problem can be quite pronounced. A ±20% variation is common when the same sample is analysed by different experimenters because their physical heights, rates and angles of sample shaking and transfer times into the magnet all affect the outcome. To combat this problem, our research team at The University of York, in collaboration with Bruker, have designed and iteratively tested an automated polarizer and sample delivery system. A communication illustrating this method employing an earlier version of the polarizer and the polarization transfer catalyst [IrCl(COD)(IMes)] (**1**) [COD = cyclooctadiene and IMes = 1,3-bis-(2,4,6-trimethyl-phenyl)imidazole-2-ylidene] has been published,^37^ and others are now adding to these early reports.^38^,^39^

In this paper, we report a series of results that were obtained when the latest version of the automated *para*hydrogen polarizer was used,^40^ which herein, we describe in greater detail. Here, we report on the use of the polarizer to investigate the biologically relevant^41^,^42^ molecule, nicotinamide (**L**) rather than the model substrates, pyridine^37^ and quinoline,^40^ that featured in our work. The importance of **L** in a clinical setting is already known; it is used as a chemo and radio sensitizer for cancer therapy,^43^ and there is evidence of it restoring cognitive deficits in Alzheimer's patients.^44^ Clearly, **L** is biologically relevant, and currently, we are working towards employing it in a first-in-man study in combination with SABRE. Understanding the type of polarized spin states and their relative amplitudes is therefore critical to this objective. The experiments employing this equipment serve to illustrate the efficiency of SABRE and provide results that dramatically improve our understanding of the physical basis of the SABRE method. This understanding has been achieved by studying the effects of the following: (i) the length of time that *para*hydrogen is bubbled through the solution present in the reaction cell (also called and henceforth referred to as the Mixing Chamber), which replaces the NMR tube described earlier in the shake/dissolve method, (ii) the pressure of *para*hydrogen gas placed over the solution and (iii) the strength of the magnetic field experienced by the sample during the polarization transfer step and the types and properties of the magnetic states that are created by SABRE. Studies of these conditions provide critical data to enable the generation of a method to optimize the level and type of ^1^H polarization created. The specific properties we deal with include an assessment of relaxation of hyperpolarized states created on nuclei in the substrate. In this context, these relaxation rates are influenced significantly subsequent to polarization transfer by the continued presence of the polarization transfer catalyst. Whilst on the one hand, the catalyst facilitates the build-up of polarization in the substrate, on the other hand, its presence creates a pseudo *T*_1_ relaxation through which the amplitudes of the hyperpolarized states are reduced. It has been reported in a theoretical paper dealing with SABRE^45^ that, in a substrate containing a two-spin system of protons, a mixture of longitudinal magnetization and longitudinal two-spin order,^46^ of the type 2*I_z_S_z_*, are created along with higher order terms.^45^ A modification of the gradient-based selection procedure called only *para*hydrogen spectroscopy (OPSY)^47^,^48^ is used to test this hypothesis by probing the predicted magnetization through the creation of appropriate zero quantum (ZQ), single quantum (SQ), double quantum (DQ), triple quantum (TQ) and quadruple quantum (QQ) longitudinal spin order terms in the hyperpolarized sample. These methods are refined to enable the assessment and quantification of the specific polarization created in **L** using SABRE. They are then used to establish strategies for the detection of ^13^C-derived magnetization.

## Experimental

### Nuclear magnetic resonance equipment

The reported NMR measurements were conducted using a Bruker 400 MHz Avance III spectrometer (Germany) equipped with a triple resonance probe with the x-coil on the outside (TXO) flow probe that was linked directly to an automated SABRE polarization system, which has been previously described.^49^ All measurements were made at 298 K.

### Automated signal amplification by reversible exchange polarization system

**Scheme 1:**
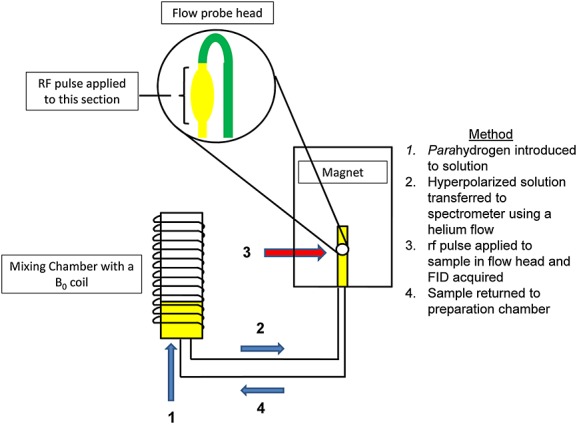
Schematic of the Polarizer, the hyperpolarization process and its subsequent NMR analysis.

The automated system (the Polarizer) is used to control the polarization transfer step. It consists of the Mixing Chamber referred to in the preceding texts where the solvent, catalyst and substrate are located. This chamber is located close to the magnet and a copper coil surrounds it, which can be used to generate a controlled magnetic environment in the Mixing Chamber that lies between −150 and 150 G. The components of the local magnetic field in which the Mixing Chamber is situated were measured using a Hall meter as follows: *x* 4.9 − 5.1 G, *y* 3.3 − 3.6 G and *z* 1.5 − 2.1 G. All magnitudes of the magnetic fields in which polarization transfer occurs [henceforth referred to as the Polarization Transfer Field (PTF)] are stated in this paper without correction for this local field. The chamber is connected to a flow probe located in the 400 MHz magnet by a liquid transfer line as shown in Scheme 1. Liquid and gas flow within the Polarizer is computer controlled from within the pulse programme through seven pneumatic valves. In this way, the process of introducing *para*hydrogen into the solution transferring the hyperpolarized material into the flow probe and acquiring these NMR data is rigorously controlled.

### Catalyst activation method

**Figure 1:**
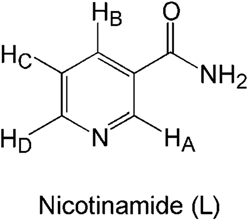
Labelled chemical structure of L.

The polarization transfer catalyst, **1**, was prepared according to a literature procedure.^50^ In a typical experiment, a deuterated methanol solution of **1** and **L** (structure depicted in Fig. 1) is purged with *para*hydrogen gas in the Mixing Chamber.

Introduction of *para*hydrogen into the Mixing Chamber is achieved by flowing *para*hydrogen through a glass frit located at the base of the Mixing Chamber. The *para*hydrogen gas is produced in a *para*hydrogen generator by cooling hydrogen gas to 30 K in the presence of an activated charcoal catalyst.^9^ In the first instance, purging the solution in the Mixing Chamber with *para*hydrogen is continued for approximately 1 min in order to activate the catalyst. During this process, 1 is converted into a dihydride complex, which contains ligated L. Helium gas is then used to shuttle the hyperpolarized solution from the Mixing Chamber to the NMR probehead where it is interrogated by appropriate NMR methods. The transportation time was calibrated to 2.9 s, and a further delay of 1.0 s was allowed for settling of the sample prior to signal acquisition. Catalyst activation is evident in the resulting ^1^H NMR spectrum as a hydride signal at δ −22.70. This is indicative of a ligand located *trans* to a hard nitrogen centre.^50^,^51^ Results detailing the characterization of the related SABRE catalyst [Ir(IMes)(pyridine)_3_(H)_2_]Cl have been communicated previously.^49^ Once catalyst activation is complete, the SABRE phenomenon can be detected by NMR spectroscopy as enhanced NMR resonances for both the free and bound L. A series of NMR measurements was conducted to explore the effect of the experimental variables described in the Introduction. This was facilitated by using the flow system to re-polarize and hence re-examine the sample as outlined in Scheme 1.

Table 1 reports the *T*_1_ values of the *z*-magnetization of the four pyridyl ring proton resonances of L at different substrate concentrations. These values were recorded both in the presence and absence of 1, whose concentration was also varied. All samples were prepared with normal H_2_ under 3 bar of pressure. It is clear from these data that the *T*_1_s of all four resonances change as the concentration of L changes. The addition of 1 to these solutions aids the *T*_1_ relaxation mechanism and results in shorter *T*_1_ values. The impact of this change on the ability of 1 to hyperpolarize L will be discussed later.

**Table 1:** *T*_1_ relaxation times for H_A_ (*I_z_*), H_B_ (*S_z_*), H_C_ (*R_z_*) and H_D_ (*T_z_*) for samples of L only at concentrations of 0.025 and 0.08 M and samples containing both L and 1 in the ratios shown (1 (2 mg) and L (0.08 M); 1 (0.2 mg) and L (0.025 M); and 1 (0.2 mg) and L (0.08 M), respectively)

Term	*T*_1_ (H_2_)/s		*T*_1_ (Cat/H_2_)/s		
		Concentration				
	[**L**] 0.08 M	[**L**] 0.025 M	[**L**] : [**1**] 16 : 1	[**L**] : [**1**] 50 : 1	[**L**] : [**1**] 160 : 1
*I_z_*	28.8	43.1	9.6	16.9	18.8
*S_z_*	13.6	11.3	6.6	7.2	9.3
*R_z_*	8.2	6.6	4.8	5.1	5.4
*T_z_*	7.2	13.5	5.4	7.9	8.8

## Results

### Signal amplification by reversible exchange of nicotinamide (L) by [IrCl(COD)(IMes) (1) and *para*hydrogen

**Figure 2:**
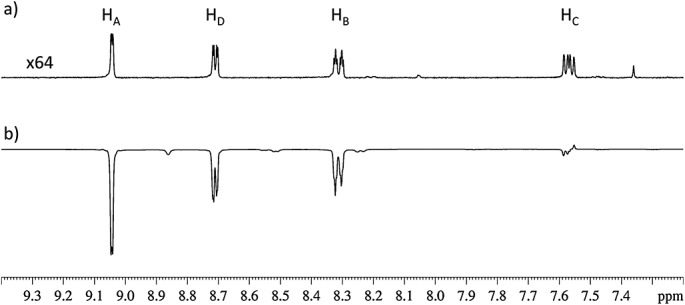
Single scan ^1^H NMR spectra of L: (a) control trace to establish normal response [×64 vertical expansion relative to (b)]; (b) after polarization transfer at a field strength of 70 G; labels H_A_–H_D_ are the positions shown in Fig. 1.

**L** is a molecule where all of the associated ^1^H and ^13^C groups are chemically inequivalent and, therefore, provide their own individual NMR responses. The spin system of the pyridyl ring ^1^H framework can be described by the spin angular momentum operators *I*, *S*, *R* and *T* labelled according to Fig. 1 with *I* ≡ H_A_, *S* ≡ H_B_, *R* ≡ H_C_ and *T* ≡ H_D_. A sample of 0.08 M of **L** and 5 mM of **1** in CD_3_OD (giving a 16 : 1 **L** to **1** ratio) was activated to enable hyperpolarization transfer from *para*hydrogen at 70 G using a 10 s *para*hydrogen purge. The ^1^H NMR spectrum obtained with a single scan 4.9 s later, after sample movement into the flow probe, is shown in Fig. 2. This NMR spectrum contains hyperpolarized signals that arise from the four ^1^H nuclei of the pyridyl ring of **L** as a consequence of SABRE.

Comparison of the single scan hyperpolarized ^1^H NMR spectrum shown in Fig. 2(b) with the corresponding thermal equilibrium trace (Fig. 2(a)) reveals that the signal for polarized H_A_ is 148 times larger than the thermal signal. Both of these ^1^H NMR spectra were collected using a *π*/2 pulse and, therefore, detect longitudinal magnetization. The corresponding signal enhancements for the protons H_D_, H_B_ and H_C_ are 105-fold, 106-fold and 7-fold, respectively. This suggests that the efficiency of polarization transfer through SABRE is highly dependent on the relative position of these protons within the molecule; a phenomenon that we have predicted theoretically to reflect the scalar coupling network of the substrate.^45^ The four smaller peaks arise from bound **L** ligands in the metal complex used as the SABRE polarization transfer catalyst. This molecule is, therefore, suitable for probing the SABRE effect and providing data with respect to the magnetic states created during the polarization transfer process.

### Characterizing the magnetic states created by signal amplification by reversible exchange polarization transfer

As already discussed, the SABRE effect creates a range of magnetic states. The proportions of these states are expected to vary with the PTF. We now describe the results we obtained from a series of measurements that probe this effect.

### Longitudinal magnetization terms created under signal amplification by reversible exchange

**Figure 3:**
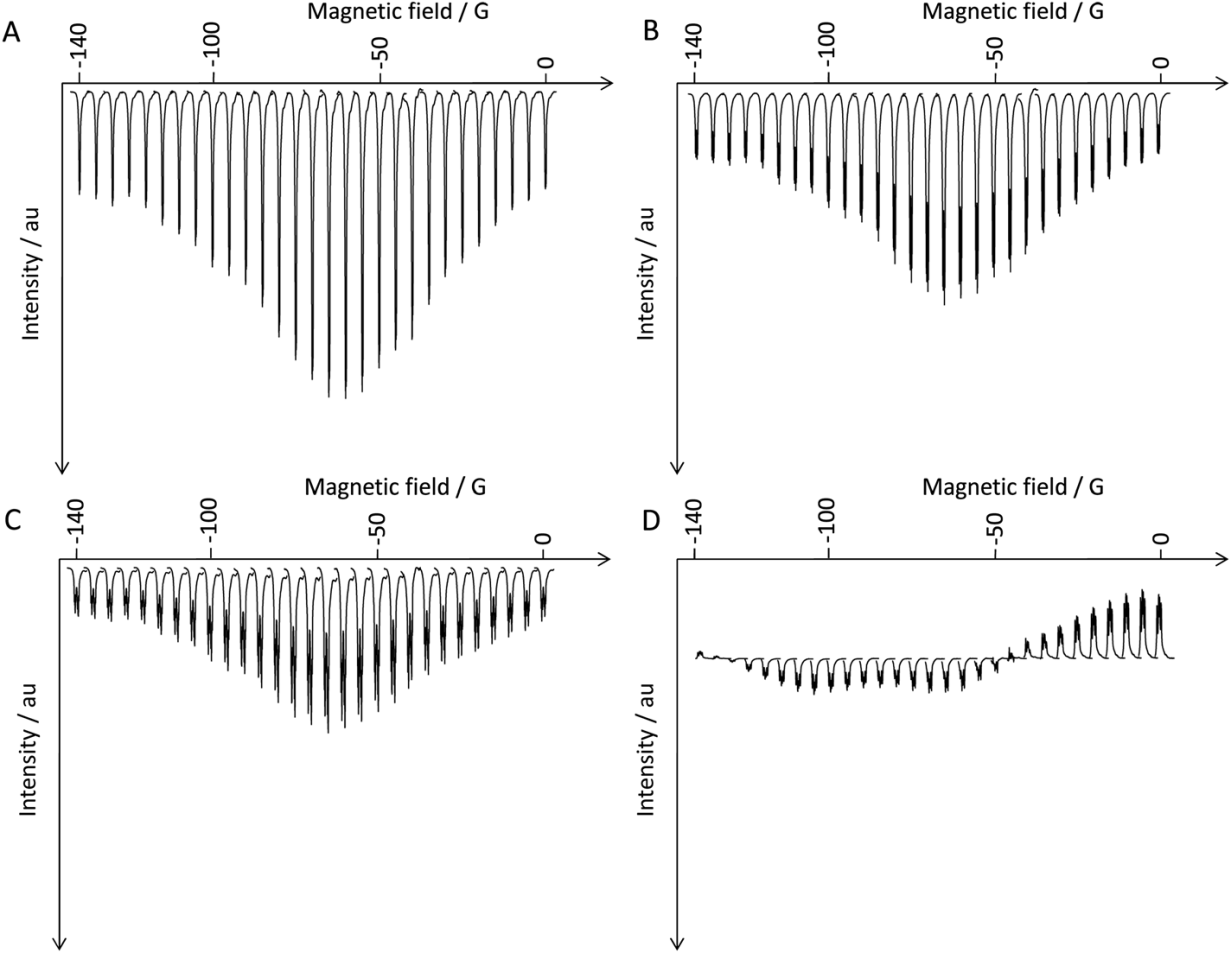
Plots of polarization transfer field versus hyperpolarized ^1^H *I_z_*, *T_z_*, *S_z_* and *R_z_* longitudinal magnetization intensity over the range 0 to −140 G (steps of 5 G) for L, determined using a *π*/2 read pulse for the signals of: (A) H_A_, (B) H_D_, (C) H_B_ and (D) H_C_ (relative amplitudes indicated).

Figure 3(a)–(d) shows how the relative amounts of *I_z_*, *S_z_*, *R_z_* and *T_z_* magnetization that are detected at each of the four proton environments (A)–(D) in **L** vary with the strength of the PTF over the range 0 to −140 G. These data were measured by applying a single *π*/2(*x*) *rf* pulse to the sample that converts, for example, in the case of proton H_A_, *I_z_* longitudinal magnetization into detectable, in-phase *I_y_* magnetization. The observed signals should not, therefore, possess any contribution from the other longitudinal terms that may be created, and to all intents and purposes, this is exactly what is seen given the potential errors in setting a *π*/2 pulse.

From these data, the maximum longitudinal magnetization-derived signal enhancement for the two *ortho* (H_A_ and H_D_) and the *para* (H_B_) sites was achieved when the PTF was approximately −70 G. These spin states in **L** are populated through the interaction with *para*hydrogen in the PTF and relax to thermal equilibrium.

### Optimization of signal amplification by reversible exchange

The effects of the magnitude of (a) the *para*hydrogen pressure, (b) the length of time for which *para*hydrogen is bubbled through the solution and (c) the concentration of **1** on the level of longitudinal magnetization that are created through SABRE are now described.

1. Effect of *para*hydrogen pressure on the level of hyperpolarization

**Figure 4:**
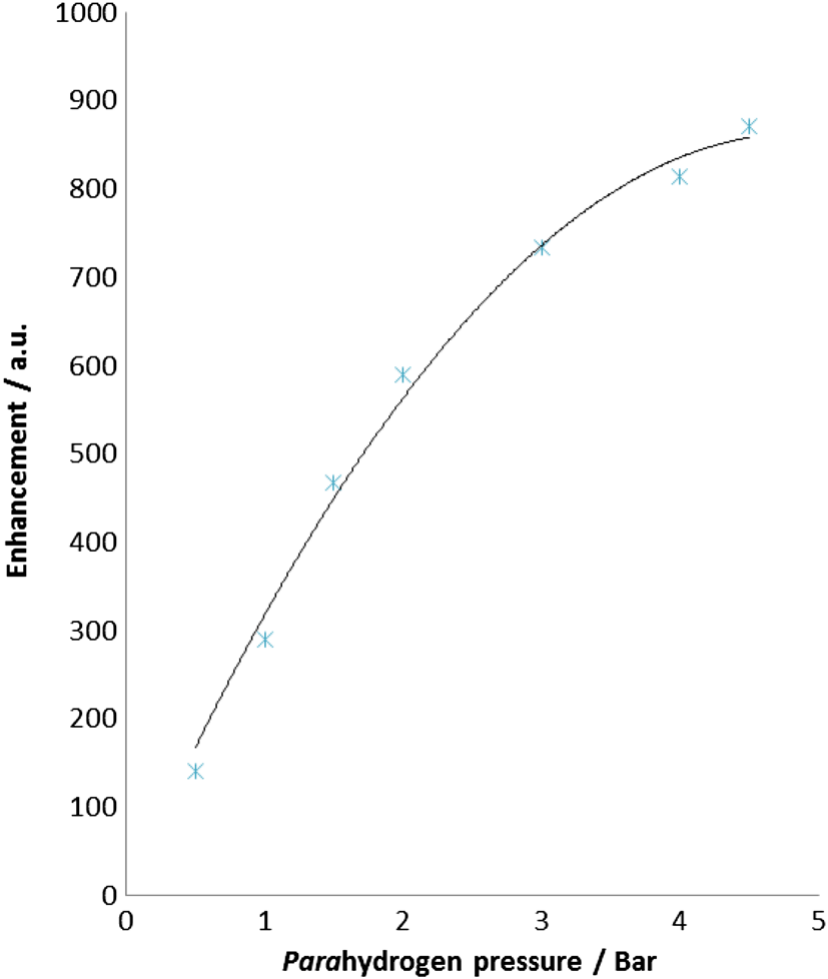
Plot of the sum of the ^1^H longitudinal magnetization NMR signal intensities, read out by a *π*/2 pulse, for the protons H_A_–H_D_ of L versus *para*hydrogen pressure.

Signal amplification by reversible exchange results from polarization that is transferred from the hydride ligands in **1**, which are derived from *para*hydrogen, to polarizable nuclei in the substrate. It might, therefore, be sensible to hypothesize that an increase in the relative concentration of *para*hydrogen will lead to greater polarization levels in the substrate. In order to probe this effect, a series of experiments was conducted in which the polarization transfer results were monitored as a function of *para*hydrogen pressure. The summed enhancement (sum of moduli of the individual enhancements) for H_A_–H_D_ is shown in Fig. 4 and confirm that increasing the concentration of *para*hydrogen in solution does indeed increase the level of detectable hyperpolarization. These measurements employed a common purge time of 6 s. Whilst at low pressure, the dependence seems to be linear, as the measurements tend to the limiting 5 bar level; there is evidence of plateauing. The propensity for multiple productive visits to the metal catalyst, and hence more productive polarization transfer, for each molecule of **L**, therefore, is increased. This is commensurate with an optimum *para*hydrogen concentration in solution, which leads to greater levels of longitudinal magnetization being created on protons H_A_–H_D_.

(b) Effect of *para*hydrogen bubbling time on the level of hyperpolarization

**Figure 5:**
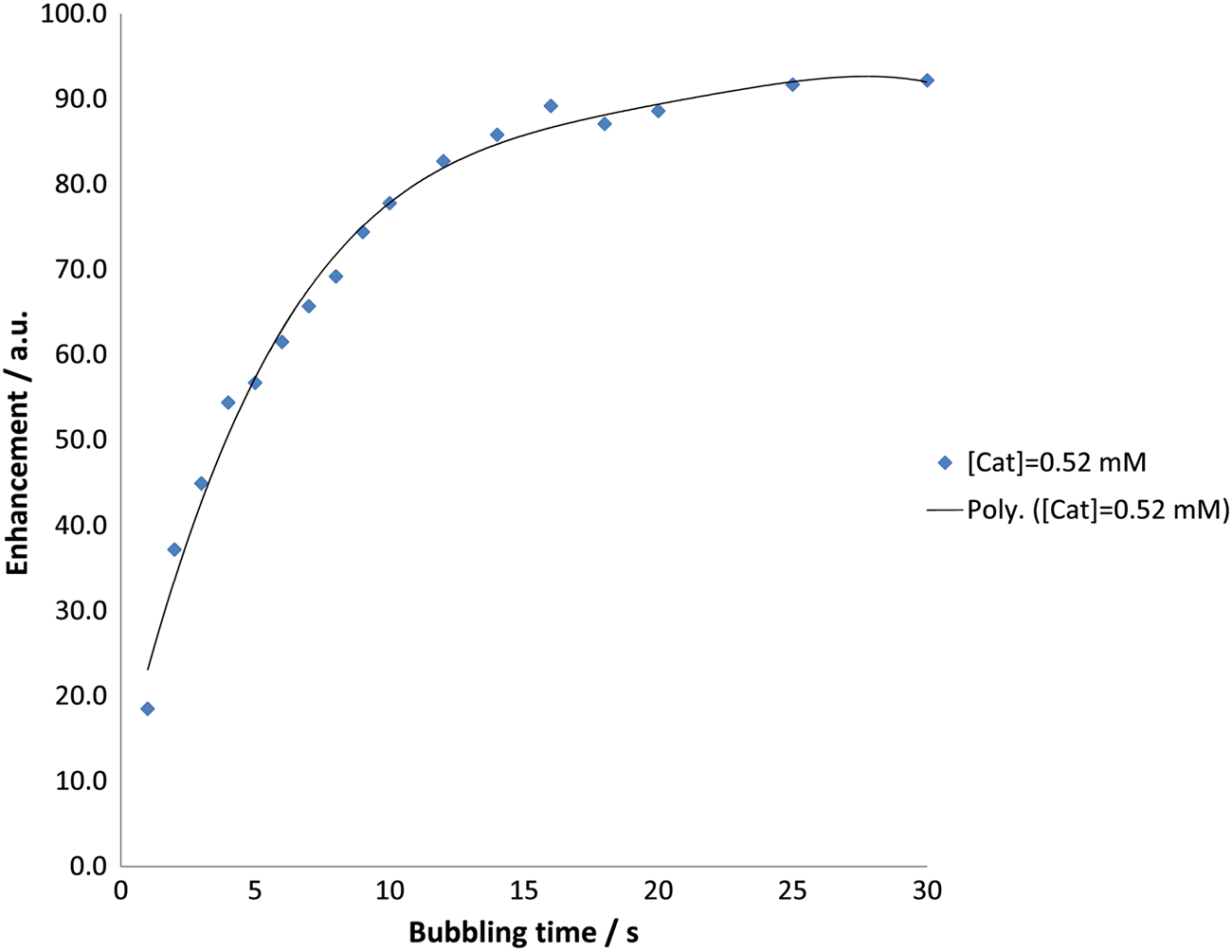
Plot showing how the *para*hydrogen bubbling time affects the strength of the hyperpolarized ^1^H NMR signal for proton H_A_ as determined using a *π*/2 read pulse and with a 1 concentration of 0.52 mM. The experimental data points and the curve of the best-fit polynomial are shown.

The number of productive ligand visits to the metal catalyst can also be increased by lengthening the total time in contact with *para*hydrogen. In order to probe this effect, the *para*hydrogen purge time was varied from 1 to 30 s. After purging, the polarized sample was transferred to the flow probe, and a *π*/2 read pulse was applied. This process is illustrated in Fig. 5 for the H_A_ resonance of **L** showing how its intensity changes with bubbling time with a catalyst concentration of 0.52 mM. Clearly, the enhancement of this signal grows in rapidly over the course of the first 10 s until it reaches a maximum enhancement value of approximately 80 relative to the thermal signal. Beyond this time, the enhancement plateaus which suggests that equilibrium has been reached wherein the rate of polarization build-up is matched by the rate of relaxation.

(c) Effect of the concentration of **1** on the level of hyperpolarization

**Figure 6:**
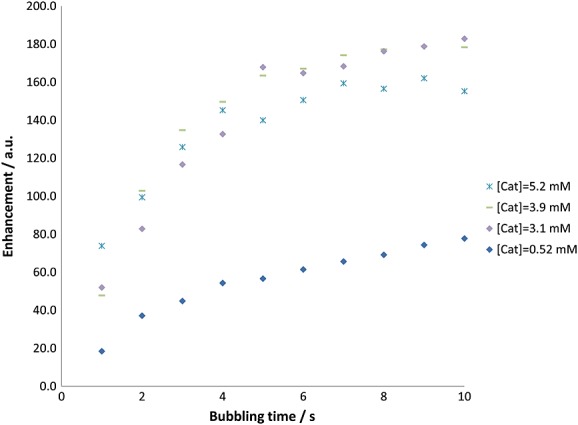
Plots showing how the *para*hydrogen bubbling time and concentration of 1 affects the strength of the hyperpolarized L ^1^H NMR signal for site H_A_ as determined using a *π*/2 read pulse.

A further set of measurements was completed in which the concentration of **1** was varied whereas the concentration of **L** remained constant. These data are shown in Fig. 6. Several further effects were evident in the associated data. The first of these is manifested in the initial growth rate, which generally increases with increasing concentrations of **1**. For the concentrations of **1** of 3.1, 3.9 and 5.2 mM, maximum enhancements were observed at bubbling times in the range 10–12 s. However, we observed that at the lower concentration of **1** of 0.52 mM, the bubbling time required to attain the maximum polarization level increased significantly. The absolute maximum was obtained with the intermediate concentration of **1** of 3.1 mM.

The rate at which polarized **L** builds-up in solution can be further evaluated by considering the *T_1_* values of protons H_A_–H_D_. Table 1 sets out these values under normal H_2_ but without O_2_, both in the presence and absence of **1**. They reveal that longitudinal relaxation is dependent upon both the concentration of **L** and **1**. The relaxation times measured in the presence of **1** were significantly shorter than those measured without it. This indicates that the catalyst not only provides a route to polarization build-up in all four protons but also a route to polarization depletion, which could be viewed as a pseudo-relaxation effect. In the Supporting Information (SI), we show that the relaxation rates of detectable two-spin and three-spin order terms are also increased. The consequence of higher concentrations of **1** during hyperpolarization transfer is that a steady-state between polarization build-up and relaxation is reached.

We conclude at this stage that it is possible to polarize **L** through SABRE and create an array of longitudinal single spin order terms whose amplitude can be increased by using an optimal PTF value, which differs according to the site. Furthermore, these amplitudes increase with increase in *para*hydrogen pressure and bubbling time. The effect of catalyst concentration is complicated by the fact that for kinetic reasons, faster transfer occurs with high concentration, but this is offset by the effect of the catalyst acting to promote relaxation of the created longitudinal terms. In order, therefore, to achieve optimal polarization, a compromise must be struck between these physical constraints.

(d) Effect of experimental parameters on the creation of higher order spin terms

We have already predicted that a range of higher order longitudinal terms should be created during the polarization transfer process. It has previously been shown that longitudinal single spin order terms can be differentiated from high order longitudinal magnetization by the application of the OPSY sequence. A series of experiments, using modified OPSY pulse sequences, illustrated in the SI, were designed to probe these terms and are described in the following sections.

**Figure 7:**
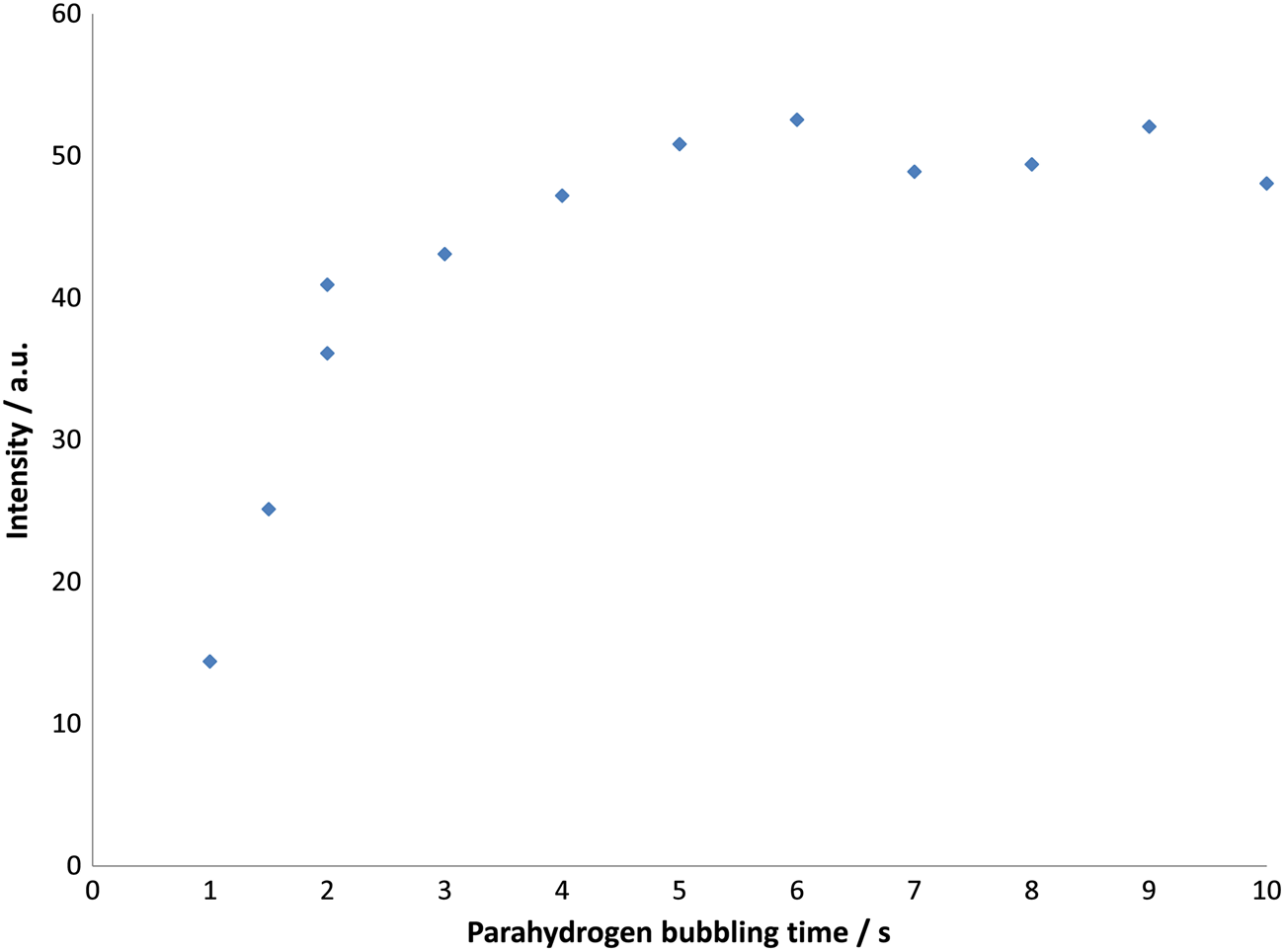
Plot showing how the *para*hydrogen bubbling time affects the strength of the hyperpolarized L^1^H *DQ* coherence NMR signal for proton H_A_ as determined using the OPSY-*d* sequence at a polarization transfer field of 65 G.

The predicted longitudinal higher order terms that are created in **L** are exemplified by 2*I_z_S_z_*, 4*I_z_S_z_T_z_* and 8*I_z_S_z_R_z_T_z_*. There are one longitudinal four-spin order, four longitudinal three-spin order and six longitudinal two-spin order terms that theoretically can be populated in **L**. In order to enable the OPSY experiment to probe a single longitudinal two-spin order term out of the set of 15 potential terms, a selective purge pulse was applied to dephase two spins (e.g. H_A_ and H_B_) whilst not affecting the other two (e.g. H_C_ and H_D_). Now when appropriate gradients are employed, this modified OPSY experiment reads out, after *rf* excitation, the signals arising from the zero and double quantum coherences that are created between H_C_ and H_D_. The curve shown in Fig. 7 follows this process as a function of bubbling time. When a 5.2 mM concentration of **1** is employed, the resulting signal attains a maximum at around 10 s before reaching a plateau in an analogous fashion to that described earlier for the longitudinal magnetization. It is possible to probe all six permutations of the longitudinal two-spin order terms associated with **L** using this approach. The relative proportions of these terms at −65 G were assessed by direct comparison using a bubbling time of 6 s. These data are presented in Table 2 and reveal that the 2*S_z_R_z_* (H_B_–H_C_) term is the most prevalent. However, with the exception of this term, enhancements of the longitudinal two-spin order terms are generally at least an order of magnitude less than the longitudinal magnetization terms. Rationalization of this can be attributed to the relatively short *T*_1_s of these higher order terms in the presence of **1** as illustrated in the SI. For example, the *I_z_R_z_* state when the concentration of **L** was 0.08 M (in the absence of **1**) was found to have a relaxation time of 3.6 s. This shortened to 2.6 s in the presence of 0.2 mg of **1** ([**L**]:[**1**] ratio of 160 : 1). It is noted that for smaller [**L**]:[**1**] ratios, the *T*_1_ values for some of the longitudinal two-spin order terms could not be easily obtained. This was most likely because of the combined effects of weak underlying signals and short *T*_1_s. For the longitudinal three-spin order terms, *T*_1_ values were even shorter; typically 1.0 s or less. Once again, some of these could not be measured within a reasonably acceptable experimental timescale. The longitudinal four-spin order term was unable to be measured, most likely because this state relaxes too quickly to obtain a value.

**Table 2:** Relative populations of the indicated states determined using in OPSY experiments for H_A_ (I_z_), H_B_ (S_z_), H_C_ (R_z_) and H_D_ (T_z_) where the signal enhancement is corrected for coherence order scaling

Product operator	Signal enhancement	Product operator	Signal enhancement	Product operator	Signal enhancement
*I_z_*	330.3	*I_z_R_z_*	32.6	*I_z_S_z_R_z_*	15.2
*S_z_*	125.0	*I_z_T_z_*	26.1	*I_z_S_z_T_z_*	3.5
*R_z_*	161.9	*S_z_R_z_*	95.8	*I_z_R_z_T_z_*	15.2
*T_z_*	238.2	*S_z_T_z_*	17.4	*S_z_R_z_T_z_*	15.1
*I_z_S_z_*	23.3	*R_z_T_z_*	50.9	*I_z_S_z_R_z_T_z_*	5.4

In these cases, the total time elapsed between transport of the sample from the Mixing Chamber into the measurement field and the time required for acquisition of the signals is often greater than at least one *T*_1_. An instantaneous transfer from low field to the measurement field would be required to measure the amplitudes of these states accurately.

We also investigated the effects of concentration on the magnitudes of the amplitudes of the six two-spin order terms. These data are shown in Table 3. For all three **L** and **1** concentration combinations, it is noted that *S_z_R_z_* consistently generates the largest signal amplitude. Reducing the concentration of **1** by a factor of 10 results in decreased amplitudes of all terms in the order of 4-fold to 10-fold. Conversely, reducing the concentration of **L** by a factor of 3.2 at the lowest concentration of **1** tested results in an approximate doubling of the amplitudes of all terms with the exception of *I_z_T_z_*, which remains broadly unchanged. We therefore conclude that the concentration dependence of the amplitudes of these longitudinal two-spin order terms is greater for variations in [**1**] than it is for variations in [**L**].

**Table 3:** Effect on the amplitudes of the six longitudinal two-spin order pairs in L after 6 s of bubbling *para*hydrogen, whilst the solution was stationed in a magnetic field of 65 G, for differing amounts of 1 and L. For each concentration of 1 and L, the relative amplitude of the signal is shown in parentheses after the amplitude

Amount of1/mg	Concentration ofL/M	*I_z_S_z_*	*I_z_R_z_*	*I_z_T_z_*	*S_z_R_z_*	*S_z_T_z_*	*R_z_T_z_*
10	0.08	23.3 (0.24)	32.6 (0.34)	26.1 (0.27)	95.8 (1.0)	17.4 (0.18)	50.9 (0.53)
1	0.08	6.6 (0.34)	5.6 (0.29)	2.9 (0.15)	19.6 (1.0)	4.3 (0.22)	12.7 (0.65)
1	0.025	11.1 (0.31)	10.0 (0.28)	2.2 (0.06)	35.3 (1.0)	7.2 (0.20)	23.0 (0.65)

The four potential longitudinal three-spin order combinations were probed in a similar way with a purge pulse applied selectively to the fourth spin. In this case, a gradient ratio of 1 : 3 was employed to select the resulting (−3) TQ coherence order associated with the excitation of this term. When the three-spin order combination, *S_z_R_z_T_z_*, (protons H_B_, H_C_ and H_D_) was examined as a function of bubbling time at a PTF of 65 G, the resulting signal reaches a maximum at around 4 s before plateauing in an analogous fashion to that described earlier in this section for the longitudinal magnetization when a 5.2 mM concentration of **1** is employed. In view of the eight coherences that are created for a three-spin order term, this sequence detects only 1/8 of the available magnetization under optimal conditions. The four-spin order state, *I_z_S_z_R_z_T_z_*, was probed using the OPSY experiment by monitoring the (−4) quadruple quantum coherence (one of the 16 potential coherences associated with this term). The amplitude of this term proved to be one of the lowest probed.

OPSY data can also be collected using broadband excitation pulses. However, it is important to note that under these conditions, all of the 15 longitudinal terms are excited. Hence, the visible signals do not correspond just to the probing of a discrete spin order, but rather arise from their sum, differentiated by the gradients according to their coherence orders. The results of this process are therefore entirely non-selective, and hence, while potential exists for internal cancellation through overlap, the rapidity with which these global excitation profiles can be collected is a significant benefit. The results of a series of these experiments are illustrated in the SI for six PTF values. The normalized, integrated signal areas associated with these measurements are listed in Table 4. These data suggest that the single quantum coherence selection filter provides the optimum detected signal. However, it can be seen that substantial levels of ZQ coherences are also present.

**Table 4:** Ratios of total signal areas for each ^1^H resonance as determined using zero (ZQ), single (SQ), double (DQ) and triple quantum (TQ) coherence selection for polarization created through a PTF of 70 G. The intensity data is normalized relative to the H_A_ signal detected in the SQ filtered experiment and the type of measurement

ZQ				SQ			
H_A_	H_D_	H_C_	H_B_	H_A_	H_D_	H_C_	H_B_
0.741	0.463	0.526	0.125	1.000	0.705	0.646	0.155
DQ				TQ			
H_A_	H_D_	H_C_	H_B_	H_A_	H_D_	H_C_	H_B_
0.016	0.025	0.031	0.038	0.002	0.004	0.005	0.006

In this section, we have detailed how SABRE creates an array of longitudinal spin orders. The amplitudes of the terms were found to follow the order single > double > triple > quadruple. Furthermore, the rate of build-up of the higher order terms proved faster than those of the single spin orders. However, as the pseudo-relaxation rates for the higher order terms are larger than those for the single spin orders, their ultimate amplitudes are smaller. The relative amplitude of these terms can, however, be controlled by the PTF. For example, the ratio of the total signal areas measured at 70 G for the H_A_ resonance in these non-selective zero, single, double and triple quantum selected experiments is 0.7400 : 1.0000 : 0.0160 : 0.0017. Given the power of NMR to characterize molecules through the probing of spin–spin couplings between groups, these observations offer significant insight into how SABRE may now be used in conjunction with multi-pulse experiments. For example, if measurements were to be completed in protio solvents, where a very strong background signal is expected, these data would suggest that selecting a ZQ coherence pathway would not only remove the solvent peaks but still enable hyperpolarized signals from **L** to be detected. This will be important if SABRE is ultimately to be used in conjunction with MRI where a strong water background is present. In the next section, we describe how SABRE polarizes **L** in a biocompatible medium.

(e) Polarization transfer to L in ethanolic solvent systems

We have also demonstrated that it is possible to polarize **L** in a biologically relevant solvent. Polarization transfer to **L** was investigated using **1** in anhydrous *d*_6_-ethanol ([**L**] : [**1**] 16 : 1). In our initial experiment, this sample was shaken in a PTF of 65 G and after transfer to the measurement field, proton H_A_ was observed to be 56-fold larger than the ethanol CDH peak at δ 3.56. In the thermal equilibrium spectrum, the ratio of the H_A_ : CDH signal integrals was 0.8 : 1.0. Subsequently, these measurements were repeated after dilution with D_2_O to produce a 50 : 50 D_2_O : *d*_6_-ethanol solution. The resulting NMR spectrum showed that H_A_ was 63-fold larger than the ethanol reference peak. In the thermal equilibrium spectrum, the ratio of the H_A_ : CDH signal integrals was 0.6 : 1.0. The overall levels of H_A_ signal enhancement are, therefore, between 70-fold and 105-fold. These lower values are a consequence of the change in the reaction kinetics of ligand exchange in these solvents. Similar signal intensity behaviour was observed in a series of analogous flow system experiments (see SI).

(f) Investigations into the ^13^C polarization created using SABRE.

**Figure 8:**
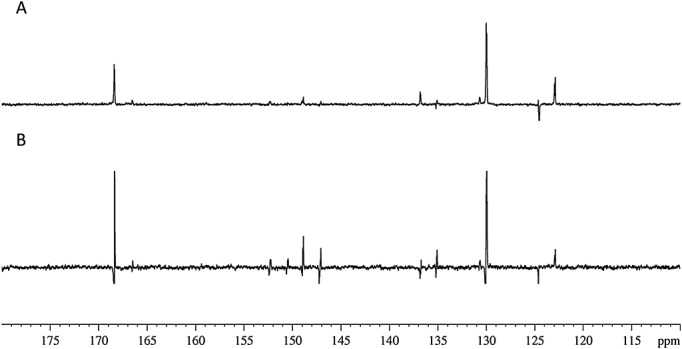
^13^C spectra acquired using a 3 ml *d*_4_-MeOD sample containing L (0.24 M) and 1 (30 mg) using a polarization transfer field of 70 G (A) and 0 G (B). Spectra are shown as a four-scan average.

We describe here a series of studies aimed at establishing that ^13^C signals in **L** can also be probed. In these investigations, the concentrations of **L** and **1** were increased threefold relative to those of the ^1^H measurements. This was necessary to increase the number of NMR active nuclei in the sample as ^13^C is only 1.1% abundant. In this part of our analysis, the operator symbols *I*, *S*, *R* and *T* are used once again but here represent the ^13^C nuclei at the C-2, C-4, C-5 and C-6 positions on the pyridyl ring of **L** (sTable 4). When a *π*/2 pulse is applied to ^13^C, only in-phase magnetization (e.g. *I_x_*) and antiphase magnetization (e.g. *I_x_S_z_* and *I_x_R_z_S_z_*) is visible. The detected signal is expected to reflect the sum of such terms according to their relative populations, which vary with the PTF. This effect is illustrated in Fig. 8 where the results of two *π*/2 excitation pulse experiments employing PTF's of 70 and 0 G are shown. The low field setting reduces the chemical shift difference between ^1^H and ^13^C nuclei such that polarization transfer is more efficient. Whilst the resulting NMR spectra contain signals that arise from all six carbon sites, the two quaternary carbon signals appear with significant intensity because of initial contributions from *z*-magnetization terms. In contrast, the three signals for the strongly proton-coupled ^13^C resonances at C-2, C-4 and C-6 appear with dominant antiphase character associated with their long-range *J_HC_* couplings under transfer at 0 G. However the *meta* C-5 signal shows antiphase character associated with its direct one-bond *J_HC_* coupling. These signals are significantly enhanced. When these experiments were conducted using a PTF of 70 G, the intensity of the observed C-5 signal increased whilst all other ^13^C resonances reduced in intensity. Consequently, varying the PTF dramatically affects the resulting ^13^C-signal intensities. A 512 scan thermal control measurement was undertaken using a 30° flip angle pulse. This resulted in only half the signal-to-noise value for the C-3 resonance when compared to that obtained in a single scan hyperpolarized spectrum using a 0 G PTF.

**Figure 9:**
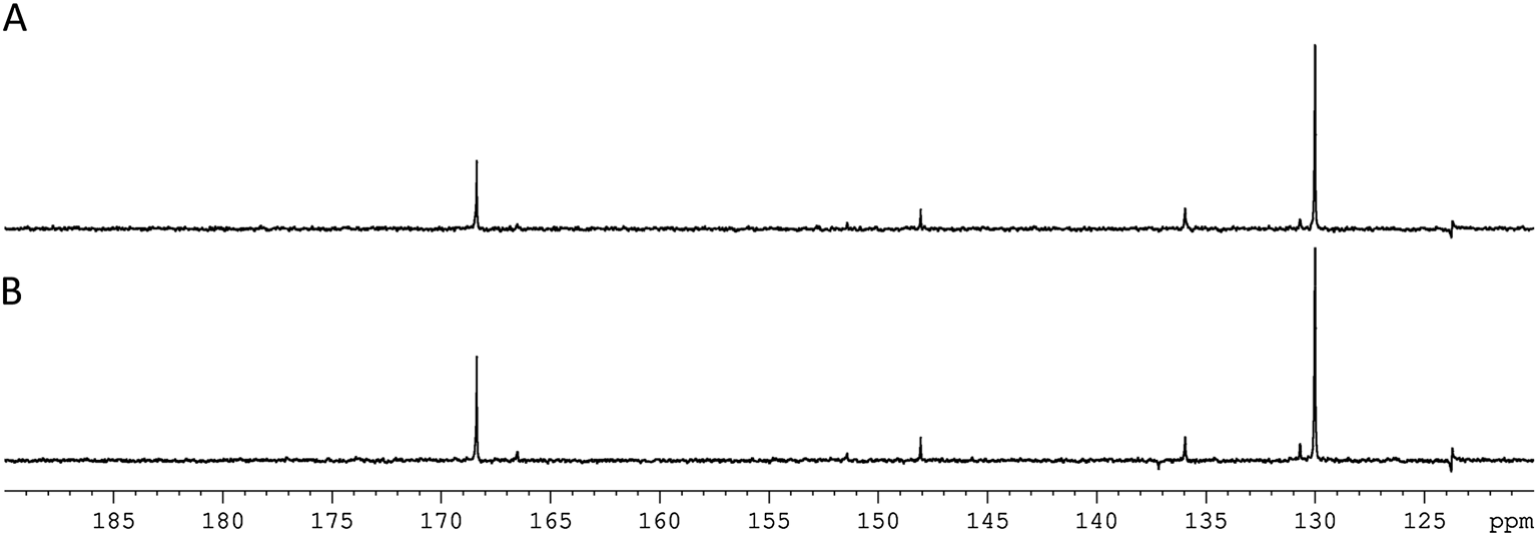
^13^C{^1^H} spectra of a 3 ml *d*_4_-MeOD sample containing L (0.24 M) and 1 (30 mg) using a refocusing delay prior to acquisition (where *J*_HC_ = 165 Hz (A) or 12 Hz (B)) and a PTF of 70 G.

Normally, ^13^C NMR spectra are recorded with ^1^H decoupling, but the antiphase character of these resonances would lead to significant signal loss in such measurements. Figure 9 illustrates how refocusing overcomes this with the six expected ^13^C-signals being observed.

**Figure 10:**
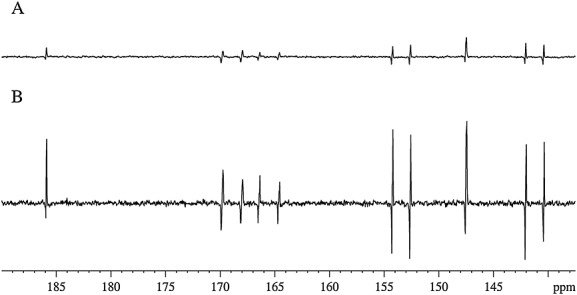
^13^C INEPTnd NMR spectra of a sample containing L (0.08 M) and 1 (10 mg) in *d_4_*-MeOD following hyperpolarization transfer at 70 G and using an evolution period of 12 Hz during the INEPT sequence. Spectra were collected using one scan (A) or four scans (B).

It is also possible to collect these ^13^C NMR spectra by polarization transfer from hyperpolarized *z*-proton states using the standard INEPT experiment without decoupling as shown in Fig. 10. Both of these NMR spectra were collected using concentrations of **L** and **1** of 0.1 M and 5.2 mM, respectively. Under these conditions, significant signal intensity is observed, and their amplitudes follow those illustrated earlier in Fig. 3 when measured as a function of the PTF. The selection of a 12 Hz coupling for refocussing proved far superior to that with 165, 64 and 8 Hz values, and in a thermal control experiment on the same sample, no discernible peaks were visible after 256 scans.

## Conclusions

In this paper, we have demonstrated that by using an integrated SABRE polarizer and flow probe, hyperpolarized nicotinamide (**L**) can be generated and interrogated using a range of NMR spectroscopic techniques. Polarization transfer from *para*hydrogen occurs predominantly to the pyridyl ring ^1^H nuclei rather than ^13^C in these measurements. Nevertheless, both ^1^H and ^13^C NMR spectra were collected which possess good lineshapes and exhibit high levels of reproducibility. We have shown that the PTF is key to achieving optimal ^1^H and ^13^C signal amplitudes. In the case of the ^1^H nuclei, optimal transfer for H_A_, H_B_ and H_D_ occurs at 65 G, whilst for H_C_, this occurs at 0 G.

The resulting ^1^H magnetization after polarization transfer is manifest in 15 detectable magnetic states, which are precisely examined here as a consequence of their first-order character. This differs from the situation in the previously studied model substrate pyridine where chemical shift equivalence and magnetic inequivalence of the *ortho-proton* and *meta*-proton spin pairs reflect a substantial challenge to the selective pulse measurements that are illustrated here and which have allowed the separation of the 15 ^1^H spin states of **L**. These states include the normal longitudinal magnetizations that are associated with the four magnetically inequivalent protons of **L**. Significantly, their amplitudes exceed those of the corresponding thermally polarized signals by between 125-fold and 330-fold for a PTF of 65 G, with the *I_z_* term associated with H_A_, the isolated *ortho*-proton, being optimally polarized. We note that H_A_ exhibits the slowest in-system relaxation with a *T*_1_ of 9.6 s whilst the *T*_1_ for H_D_ is 5.4 s. The order of the rates of relaxation is H_C_ > H_D_ > H_B_ > H_A_. The ratio of the enhancements H_A_/H_D_ is 1.4 (330.3/238.2) whilst that of their *T*_1_s is 1.8 thereby confirming that the *I_z_* state for H_A_ is the most optimally populated state. In contrast, the signal for H_B_ possesses the lowest amplitude of the four longitudinal magnetization states even though its relaxation time is larger than those of either H_C_ or H_D_. Hence, we can conclude unambiguously that SABRE transfer into H_B_ is less efficient than that into the other sites. Given that transfer into H_A_ and H_D_ is also more efficient than transfer into H_B_, we can also conclude that transfer efficiency proceeds according to H_A_ > H_D_ > H_C_ > H_B_ under SABRE for the longitudinal magnetization states of the ring protons of **L**. We note that Fig. 3 reveals a phase change in the spectrum associated with proton H_c_, and we are currently investigating the origin of this effect. Ivanov and co-workers have postulated a role for level anti-crossings in spectral phase changes.^52^

Furthermore, we have detected all six of the longitudinal two-spin order terms that might be expected. These proved to be created more rapidly than the longitudinal magnetization states, but their more rapid relaxation rates led to lower observed amplitudes at the point of measurement. This illustrates just how important it will be in the future to separate the SABRE catalyst from the substrate because it has been shown here to both create the hyperpolarization in **L** and subsequently destroy it through further interaction (referred to here as pseudo-relaxation). We are currently working to understand how deuterium labelling affects these pseudo-relaxation rates.

Whilst the creation of four three-spin and one four-spin longitudinal order terms has been demonstrated experimentally through their direct detection, albeit with smaller amplitudes, their relaxation times are even smaller. We used a version of the OPSY^47^,^48^ sequence where shaped pulses were used in conjunction with gradients to select specific states, and subsequently identify and quantify them in hyperpolarized **L**. A version of this pulse sequence was similarly used to create these states in the absence of *para*hydrogen in order measure their *T*_1_s. Experimentally, we found it impractical to produce some of the higher order states in these control measurements, probably because of their short relaxation times. However, we note that they could be observed and distinguished through SABRE. This situation is not surprising because it should be realized that these additional states are not populated in normal thermally equilibrated systems. Their amplitudes, which we have quoted relative to those of hyperpolarized longitudinal magnetization states for comparison purposes, are in reality dramatically larger than what they would be in a thermal equilibrium experiment. As a consequence, one route to using SABRE in the future may be to address normally inaccessible states.

Further to this, we have shown that it is possible to control the relative amplitudes of these states by varying the concentration of *para*hydrogen in solution and the concentrations of both the catalyst and substrate. Additionally, we have demonstrated that the continued bubbling of *para*hydrogen through a solution in the Mixing Chamber increases the level of polarization up to a maximum level beyond which the effects of relaxation appear to result in the establishment of an equilibrium within the hyperpolarized spin-state manifold. Therefore, these observations collectively serve to illustrate ways in which the SABRE experiment can be controlled. This could facilitate achievement of the optimum starting points for subsequent NMR/MRI measurements where specific initial states of magnetization are desired.

There is currently great interest in using hyperpolarization methods to prepare contrast agents for use in MRI. Here, we have illustrated that whilst SABRE is not without its complexities, these can be understood through the application of a series of relatively simple NMR procedures and validate the states that have been theoretically predicted are indeed formed. We have also illustrated here that it is possible to hyperpolarize biologically relevant **L** in an ethanol–water solvent system, which after dilution, would be suitable for subsequent *in vivo* measurements.

A series of ^13^C NMR spectra have extended these observations. We have described our results of monitoring SABRE polarized **L**, both with and without proton decoupling. These NMR spectra confirm that a further series of two-spin order terms can again be readily created, but now shared between ^1^H and ^13^C nuclei. The probing of these states results in the observance of antiphase ^13^C signals with substantial amplitudes and serves to demonstrate the potential of this method for the rapid collection of fully coupled ^13^C data. Whilst decoupling of these NMR spectra was achieved by refocusing, there is a resulting drop in the signal-to-noise ratios of the quaternary ^13^C-signals although those for the CH groups are increased. We have also shown that the resulting hyperpolarized fully coupled INEPT spectra exhibit further improvements in signal-to-noise. This is reflected in the fact that the efficiency of the INEPT experiments parallels that of hyperpolarization transfer under SABRE to the proton *z*-terms. Hence, a careful consideration of the desired proton spin order needs to be made, with the PTF set accordingly, if optimal ^13^C measurements are to be made.
