# Supplementary material for: Probing signal amplification by reversible exchange using an NMR flow system
Source: Magn Reson Chem. 2014 May 6;52(7):358–69. doi: 10.1002/mrc.4073 (PMC4190690; doi:10.1002/mrc.4073)
Supplement: Supplementary file 1 [file mrc0052-0358-sd1.docx]

Probing Signal Amplification by Reversible Exchange using an NMR flow system

Ryan E. Mewis^a^, Kevin D. Atkinson^a^, Simon B. Duckett^a†^, Michael J. Cowley^a^,Gary G. R. Green^a^, Richard A. Green^a^, Louise A. R. Highton^a^, Lyrelle S. Lloyd^a^, David Kilgour^b^, Joost A. B. Lohman^c^ and David C. Williamson^a^.

^a^Centre for Hyperpolarisation in Magnetic Resonance, University of York, Heslington, York, YO10 5DD, UK. Tel: +44 1904 322564; E-mail: [simon.duckett@york.ac.uk](mailto:simon.duckett@york.ac.uk).

^b^Bruker BioSpin GmbH, Silberstreifen 4, 76287 Rheinstetten, Germany

^c^Bruker UK, Limited, Banner Lane, Coventry, CV4 9GH,UK

**Supplementary Information**

[1. General Considerations 3](#_Toc380576824)

[2. Schematic representation of the Polarizer 4](#_Toc380576825)

[3. Summary of the experimental methods used for magnetic state interrogation. 5](#_Toc380576826)

[4. Calculation of Enhancement Factors 6](#_Toc380576827)

[5. *T*_1_ data for the longitudinal one-, two-, three- and four-spin order in L 7](#_Toc380576828)

[6. Graph showing the build-up of the ZQ, DQ, TQ and QQ coherence terms 9](#_Toc380576829)

[7. Graph showing the build-up of the TQ coherence term for H_B_H_C_H_D_ 10](#_Toc380576830)

[8. Effect of parahydrogen pressure on the detected ^1^H longitudinal magnetization NMR signal intensities 11](#_Toc380576831)

[9. 2D plot showing the association of ZQ coherence 12](#_Toc380576832)

[10. Effect of PTF versus measured ^1^H coherence level 13](#_Toc380576833)

[11. Plot of amplitudes of the six longitudinal two-spin order pairs of L after hyperpolarization at varying PTFs 14](#_Toc380576834)

[12. sFigure 7: Modified OPSY sequences used as part of this work 16](#_Toc380576835)

[13. ^13^C chemical shifts of L 18](#_Toc380576836)

[14. ^1^H NMR measurement under SABRE in a 50:50 ethanol:D2O mixture using the flow apparatus. 18](#_Toc380576837)

# General Considerations

**1** was prepared according to a literature procedure.[^1^](#_ENREF_1) In a typical experiment, a deuterated methanol solution of **1** and **L** (Figure 1) is purged with *para*hydrogen in the Mixing Chamber. *Para*hydrogen is introduced into the Mixing Chamber through a glass frit located at its base. The *para*hydrogen is produced in a generator by cooling hydrogen gas to 30 K in the presence of an activated charcoal catalyst.[^2^](#_ENREF_2) At first, purging the solution in the Mixing Chamber with *para*hydrogen is continued for approximately 60 s in order to activate the catalyst. Liquid and gas flow within the Polarizer is computer-controlled through seven pneumatic valves via the pulse program.Thus, introducing *para*hydrogen into the solution, transferring the hyperpolarized sample into the flow probe and acquiring NMR data is rigorously controlled.

**NMR Equipment.** NMR measurements were made using a Bruker 400 MHz Avance III spectrometer equipped with a TXO flow probe linked directly to the Polarizer.[^3^](#_ENREF_3) All measurements were made at 298 K.

# Schematic representation of the Polarizer


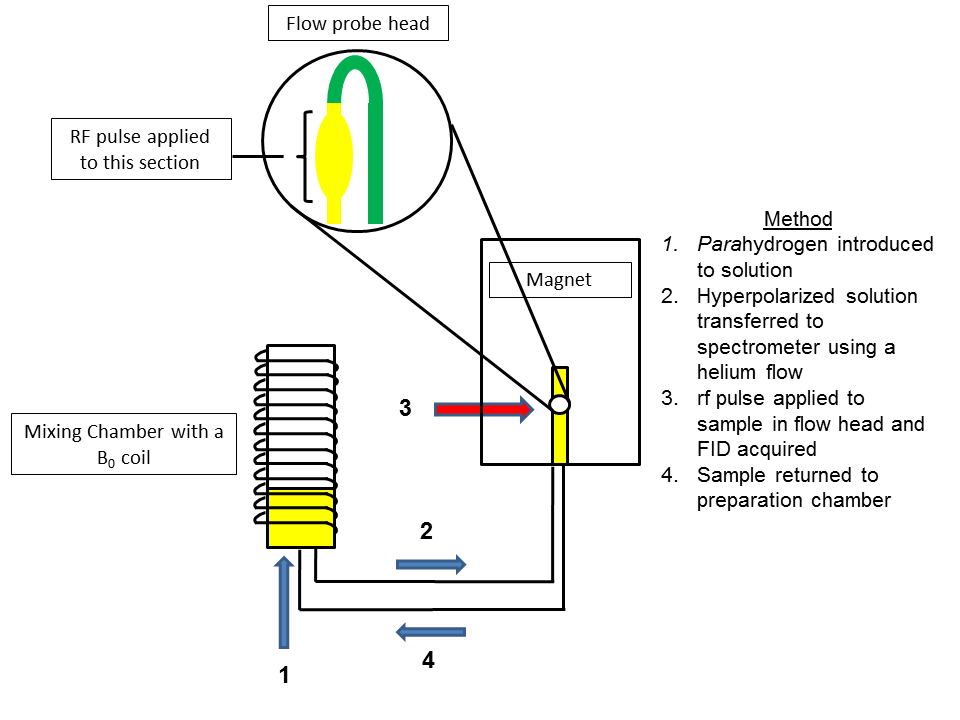


sScheme 1 : Schematic of the Polarizer, the hyperpolarization process and its subsequent NMR analysis.

# Summary of the experimental methods used for magnetic state interrogation.

It has been suggested previously that the SABRE process generates states that are associated with longitudinal magnetization, longitudinal two-spin order and higher-order longitudinal terms reflecting the finite size of the spin system,[^4^](#_ENREF_4) with *ZQ_x_* and related coherences averaging to zero over the time of the reaction. The persistent longitudinal spin order terms are probed using *rf* pulses in conjunction with magnetic field gradients according to their coherence levels in order to assess their magnitudes.

When a simple two-spin system, possessing longitudinal two-spin order, 2*I*_z_*S*_z_, is considered, both zero and double quantum coherences are created following a radiofrequency pulse and can subsequently be probed. This situation arises because the 2*I*_y_*S*_y_ coherent state results after a *π*/2(*x*) *rf* pulse which is a mixture of zero and double quantum coherences (*ZQ_x_* – *DQ_x_*). These two coherences can be differentiated because *DQ_x_* (*I*_x_*S*_x_ - *I*_y_*S*_y_) evolves at the frequency *ω_I_* + *ω_s_* whilst *ZQ_x_* evolves at *ω_I_* - *ω_s_*. The resulting *DQ_y_* (*I*_y_*S*_x_ + *I*_x_*S*_y_) and *DQ_y_* (*I*_y_*S*_x_ - *I*_x_*S*_y_) coherences provide the observable magnetization after a second *π*/2(*y*) *rf* pulse via the 2*I*_y_*S*_z_ and 2*I*_z_*S*_y_ terms which are produced. The necessary evolution time is encoded by a short time period prior to the second *rf* pulse. The double quantum coherence pathway is selected when a pair of gradient pulses, in the ratio 1:2, sandwich the second *π*/2 *rf* pulse. When the gradient ratio is set to 1:0, the zero quantum coherence pathways are selected. There are six such two-spin order terms associated with the four proton spins of nicotinamide. These can be probed selectively if spoiler pulses are included to dephase magnetization associated with the two spins whose chemical shifts lie outside the region of the chemical shifts of the selected pair.

When the gradient ratio is set at 1:1, single quantum coherence is selected and in-phase *x*-magnetization is observed. This is created from longitudinal magnetization, four of which such states exist for the protons in this molecule. Unfortunately, after *rf* excitation, there are further single quantum coherence pathways associated with the four longitudinal three-spin order terms that are predicted. However, these combinations can again be differentiated by the addition of appropriate purge pulses. The single longitudinal four-spin order term that is predicted also yields zero, double and quadruple coherence orders after excitation. This term is uniquely selected when a 1:4 gradient ratio is applied.[^4^](#_ENREF_4) The relative sensitivity of these experiments is controlled by the number of coherences that are created and observed. For example, there are two triple quantum coherences (±3) and six single quantum coherences (three each of ±1) associated with the three-spin coherences. The relative sensitivity of this method is thus one in eight. In the case of the four-spin coherences*,* six zero quantum, eight double quantum and two quadruple quantum pathways exist. The relative sensitivity of these measurements must, therefore, be considered carefully with their optimal detection. This suggests that the measured signal intensities must be multiplied by 2, 4, 16 and 32 for the longitudinal magnetization*,* longitudinal two-spin order*,* longitudinal three-spin order longitudinal four-spin order terms, respectively.

# Calculation of Enhancement Factors

For calculation of the enhancement of 1H NMR signals the following formula was used

*E* = enhancement

*S_pol_* = signal of polarized signal

*S_unpol_* = signal of the unpolarized (reference) sample

Experimentally, the reference spectra were acquired with the same sample that was used for the hyperpolarisation measurement after it had fully relaxed (typically 5-10 minutes at high magnetic field). Reference and polarized spectra were collected using identical acquisition parameters, particularly the receiver gain. The raw intergrals of the relevant resonances in the polarized and unpolarized spectra were used to determine the enhancement level.

# *T*_1_ data for the longitudinal one-, two-, three- and four-spin order in L

sTable 1: *T*_1_ Relaxation times for longitudinal one-, two-, three- and four- spin order terms of a sample containing 1 (2 mg) and L (0.08 M)

| Term | *T*_1_ (H_2_) /s | *T*_1_ (Cat/H_2_) /s | Term | *T*_1_ (H_2_) /s | *T*_1_ (cat/H_2_) /s | Term | *T*_1_ (H_2_) /s | *T*_1_ (cat/H_2_) /s |
| --- | --- | --- | --- | --- | --- | --- | --- | --- |
| *I_z_* | 36.2 | 9.6 | *I_z_R_z_* | 3.61 | nm | *I_z_S_z_R_z_* | 1.46 | 0.82 |
| *S_z_* | 10.4 | 6.6 | *I_z_T_z_* | nm | nm | *I_z_S_z_T_z_* | nm | Nm |
| *R_z_* | 6.4 | 4.8 | *S_z_R_z_* | 5.33 | 2.34 | *I_z_R_z_T_z_* | nm | Nm |
| *T_z_* | 12.0 | 5.4 | *S_z_T_z_* | 3.62 | 1.87 | *S_z_R_z_T_z_* | 1.24 | 0.99 |
| *I_z_S_z_* | 4.14 | 1.41 | *R_z_T_z_* | 5.34 | 1.45 | *I_z_S_z_R_z_T_z_* | nm | Nm |

nm = not measured

sTable 2: *T*_1_ Relaxation times for longitudinal one-, two-, three- and four- spin order terms of a sample containing 1 (0.2 mg) and L (0.08 M)

| Term | *T*_1_ (H_2_) /s | *T*_1_ (Cat/H_2_) /s | Term | *T*_1_ (H_2_) /s | *T*_1_ (cat/H_2_) /s | Term | *T*_1_ (H_2_) /s | *T*_1_ (cat/H_2_) /s |
| --- | --- | --- | --- | --- | --- | --- | --- | --- |
| *I_z_* | 36.2 | 18.8 | *I_z_R_z_* | 3.61 | 2.60 | *I_z_S_z_R_z_* | 1.46 | 0.81 |
| *S_z_* | 10.4 | 9.3 | *I_z_T_z_* | nm | nm | *I_z_S_z_T_z_* | nm | nm |
| *R_z_* | 6.4 | 5.4 | *S_z_R_z_* | 5.33 | 3.19 | *I_z_R_z_T_z_* | nm | nm |
| *T_z_* | 12.0 | 8.8 | *S_z_T_z_* | 3.62 | 3.75 | *S_z_R_z_T_z_* | 1.24 | 0.99 |
| *I_z_S_z_* | 4.14 | 3.36 | *R_z_T_z_* | 5.34 | 2.96 | *I_z_S_z_R_z_T_z_* | nm | nm |

nm = not measured

sTable 3: *T*_1_ Relaxation times for longitudinal one-, two-, three- and four- spin order terms of a sample containing 1 (0.2 mg) and L (0.025 M)

| Term | *T*_1_ (H_2_) /s | *T*_1_ (Cat/H_2_) /s | Term | *T*_1_ (H_2_) /s | *T*_1_ (cat/H_2_) /s | Term | *T*_1_ (H_2_) /s | *T*_1_ (cat/H_2_) /s |
| --- | --- | --- | --- | --- | --- | --- | --- | --- |
| *I_z_* | 43.1 | 16.9 | *I_z_R_z_* | nm | nm | *I_z_S_z_R_z_* | nm | nm |
| *S_z_* | 11.3 | 7.2 | *I_z_T_z_* | nm | nm | *I_z_S_z_T_z_* | nm | nm |
| *R_z_* | 6.6 | 5.1 | *S_z_R_z_* | nm | 1.08 | *I_z_R_z_T_z_* | nm | nm |
| *T_z_* | 13.5 | 7.9 | *S_z_T_z_* | nm | nm | *S_z_R_z_T_z_* | nm | nm |
| *I_z_S_z_* | nm | nm | *R_z_T_z_* | nm | 0.91 | *I_z_S_z_R_z_T_z_* | nm | nm |

nm = not measured

N.B. Some terms were not measured due to insufficient signal-to-noise such that they could not be detected on an appropriate timescale.

# Graph showing the build-up of the ZQ, DQ, TQ and QQ coherence terms


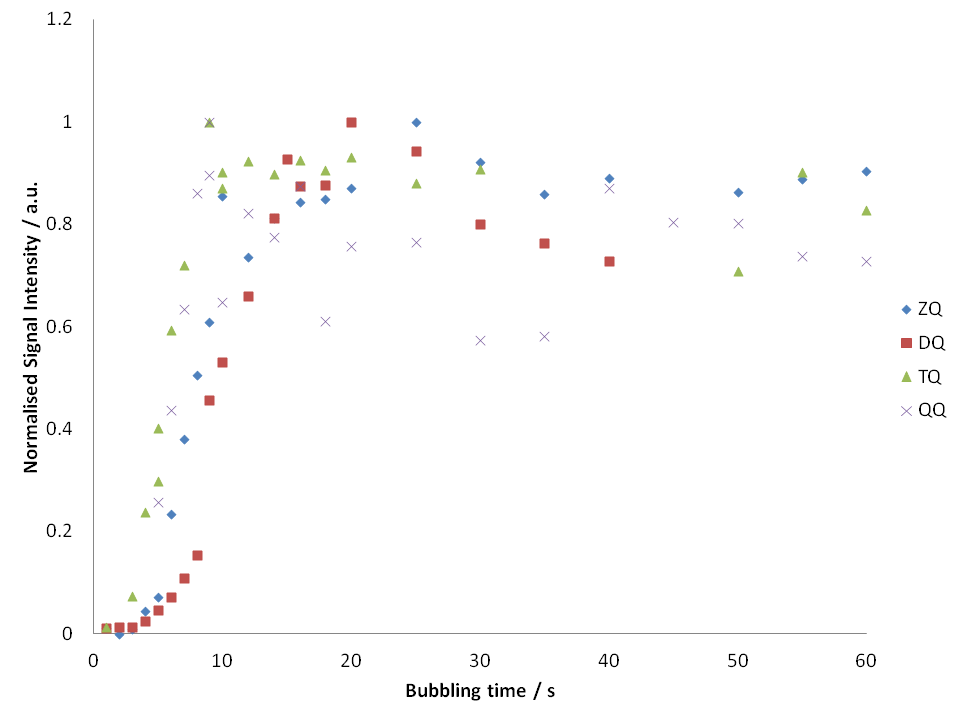


sFigure 1: Graph showing the build-up of the ZQ, DQ, TQ and QQ coherences for as a function of *para*hydrogen bubbling time at a PTF of 65 G.

# Graph showing the build-up of the TQ coherence term for H_B_H_C_H_D_


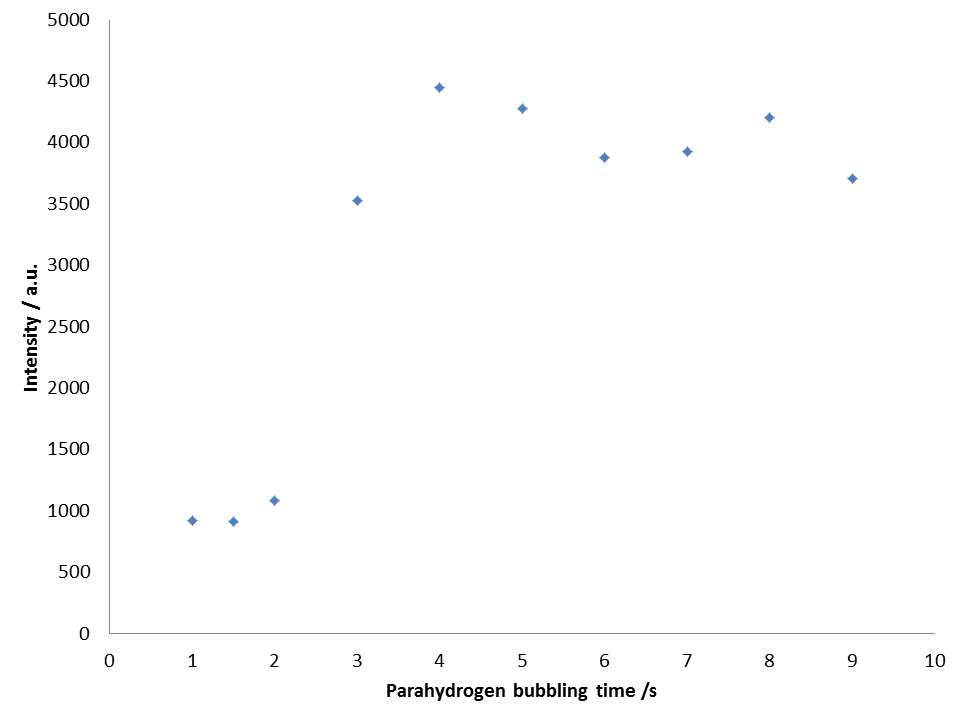


sFigure 2: Graph showing the build-up of the TQ coherence for H_B_-H_C_-H_D_ as a function of *para*hydrogen bubbling time at a PTF of 65 G.

# Effect of parahydrogen pressure on the detected ^1^H longitudinal magnetization NMR signal intensities


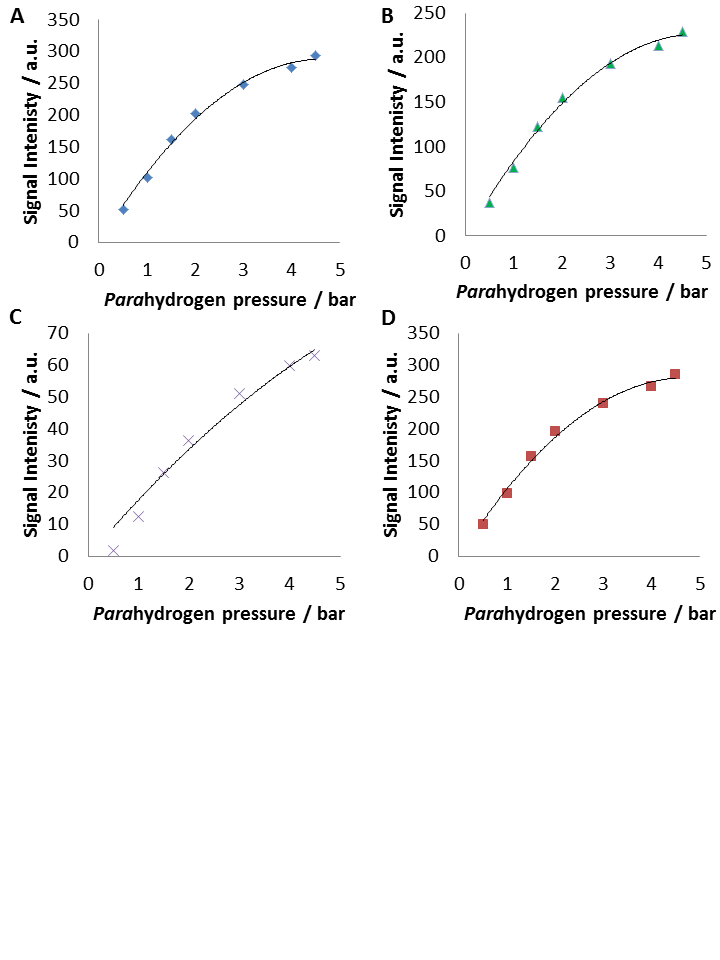


sFigure 3: *Para*hydrogen purge pressure effects on the ^1^H longitudinal magnetization NMR signal intensities, read out by a *π*/2 pulse, for the protons H_A_-H_D_, respectively, of L

# 2D plot showing the association of ZQ coherence

In this measurement, two *π*/2 pulses are employed and are separated by a delay to allow magnetization encoding as described in the Experimental section. The delay can be incremented in an analogous way to that of a COSY measurement. The resulting 2D dataset, after Fourier transformation in the second dimension, produces cross peaks at resonance positions associated with linked zero quantum coherences between protons H_A_ and H_C_. The 2D spectrum is shown in sFigure 9.


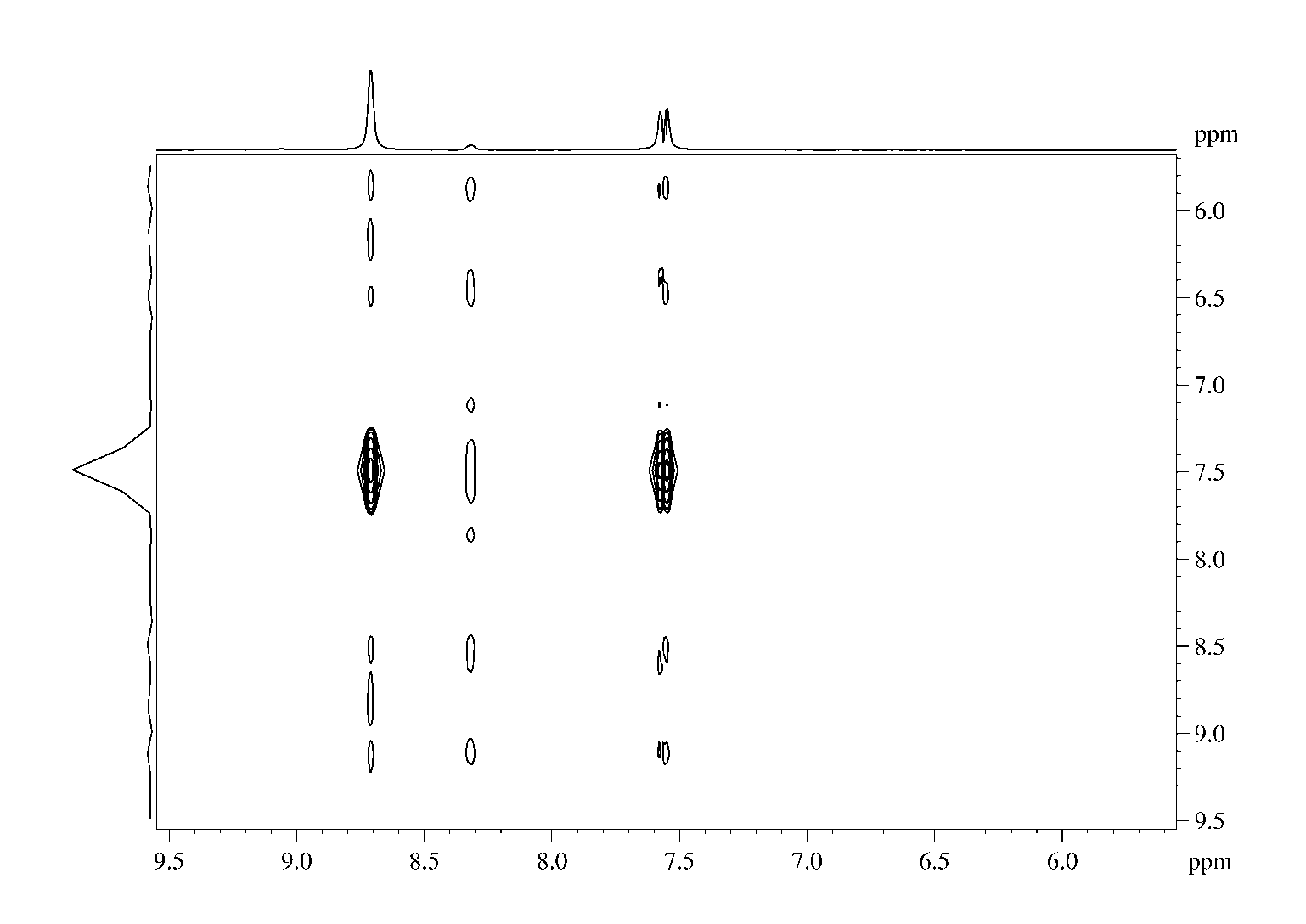


sFigure 4: 2D plot showing the resonances associated with zero quantum coherence.

# Effect of PTF versus measured ^1^H coherence level


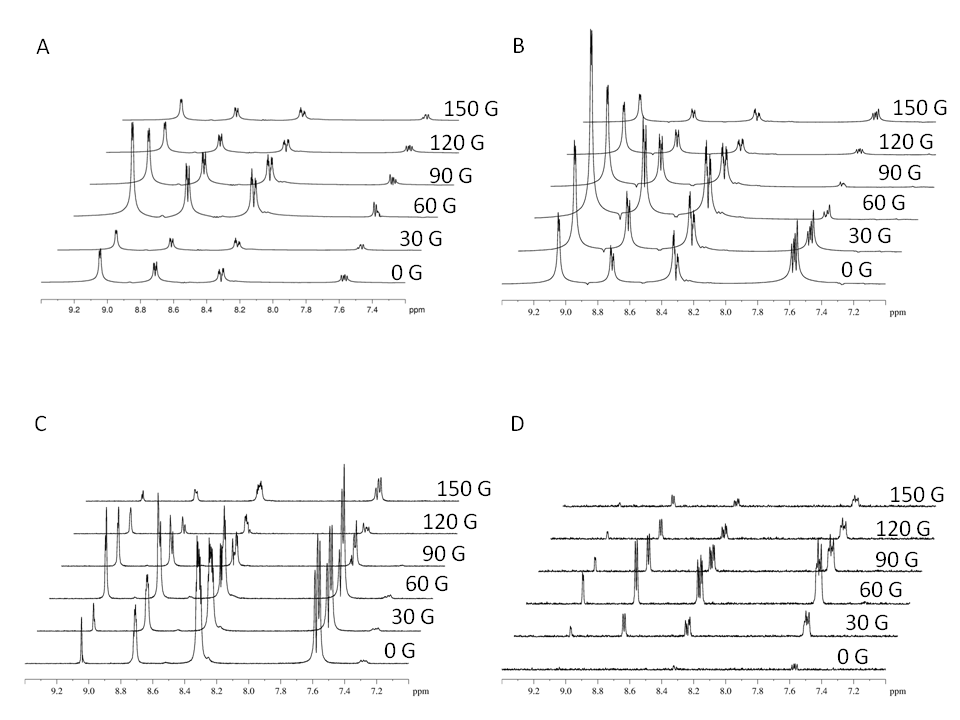


sFigure 5 : Plots showing effect of the PTF versus measured hyperpolarized ^1^H coherence level over the range 0 – 150 G (in steps of 30 G) for the four aromatic protons of L determined in the non-selective OPSY experiment for: (a) zero, (b) single, (c) double and (d) triple quantum coherence pathways.

# Plot of amplitudes of the six longitudinal two-spin order pairs of L after hyperpolarization at varying PTFs

**
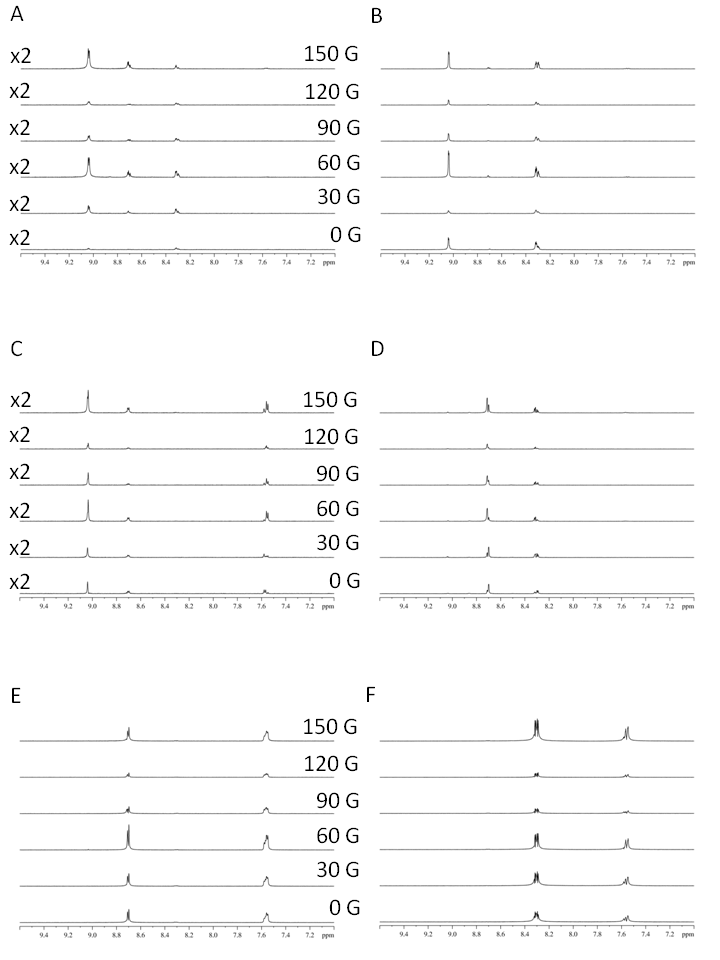
**

sFigure 6: PTF versus detected hyperpolarized ^1^H coherence intensity plots for L at field values 0, 30, 60, 90, 120 and 150 G. The spectra were acquired using a modified OPSY-*d* experiment in which both *π*/2 pulses were replaced by two selective *π*/2 pulses applied to resonances at: (A) 9.04 (H_A_) and 8.71 (H_D_); (B) 9.04 (H_A_) and 8.31 (H_B_); (C) 9.04 (H_A_) and 7.57 (H_C_); (D) 8.71 (H_D_) and 8.31 (H_B_); (E) 8.71 (H_D_) and 7.57 (H_C_); and (F) 8.31 (H_B_) and 7.57 (H_C_) (spectrum shown using an x8 vertical expansion relative to all other traces).

The appearance of a third peak in spectra A-C is because bound resonances are excited by the selective pulse (due to them being very close in chemical shift to the excited resonances). During the course of the acquisition, bound molecules responsible for these resonances become free molecules and therefore have a frequency shift in terms of their resonances. Signals are thus observed for sites that were not excited due to exchange.

# sFigure 7: Modified OPSY sequences used as part of this work


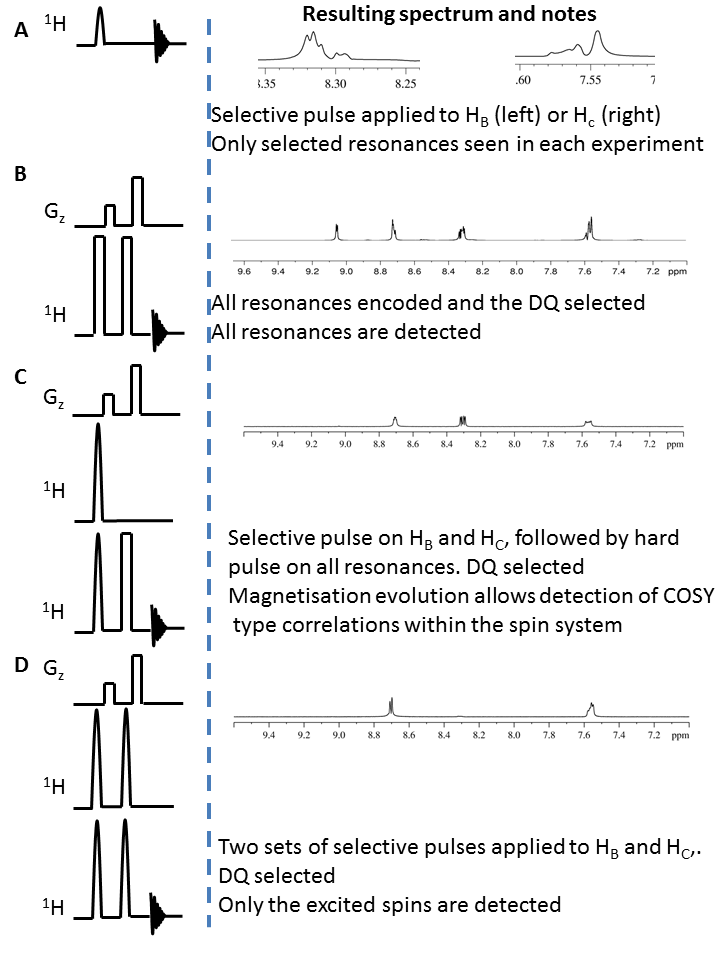


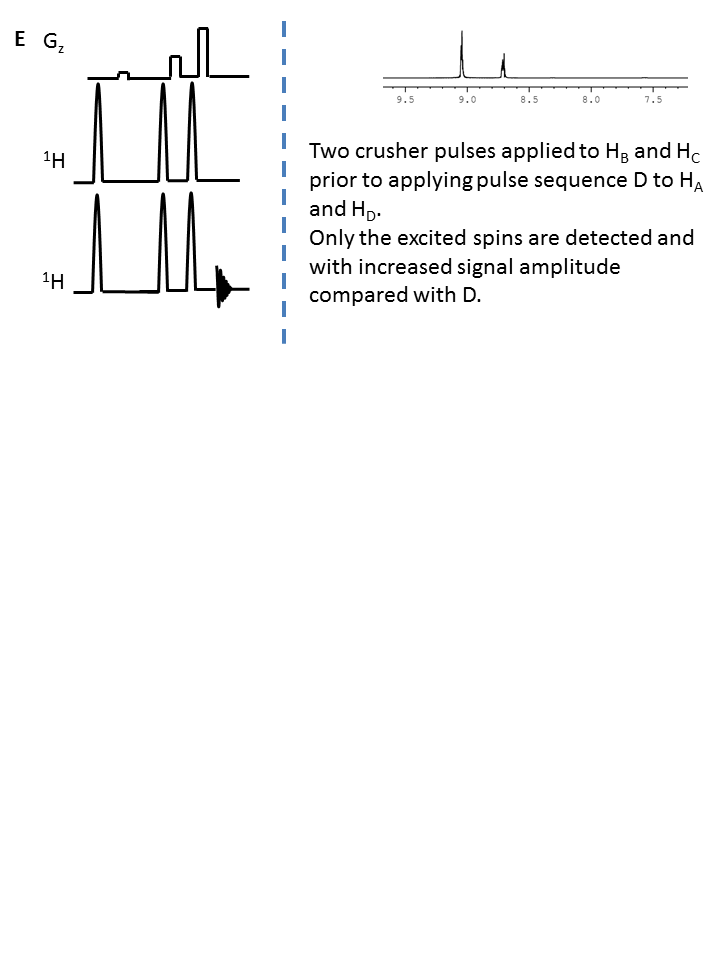


# ^13^C chemical shifts of L

sTable 4: ^13^C chemical shifts of L

| Carbon position | Chemical shift (ppm) |
| --- | --- |
| 2 | 151.47 |
| 3 | 130.44 |
| 4 | 136.01 |
| 5 | 123.77 |
| 6 | 148.08 |
| 7 | 167.49 |

# ^1^H NMR measurement under SABRE in a 50:50 ethanol:D_2_O mixture using the flow apparatus.


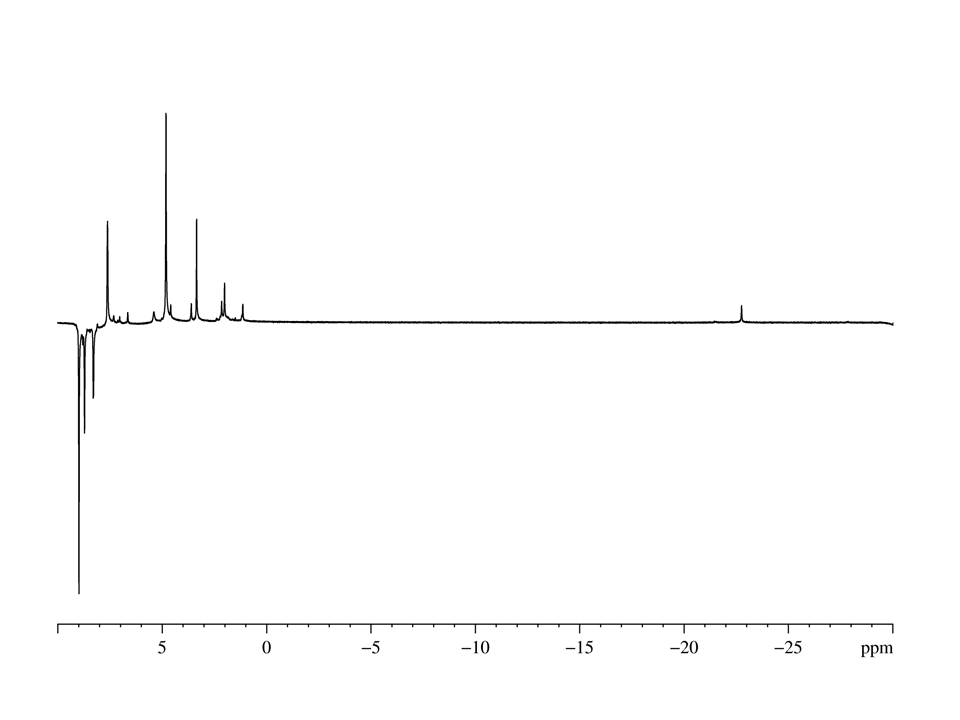


sFigure 8: Hyperpolarized ^1^H NMR spectrum obtained on a ethanol : D_2_O mixture (50:50) in the flow system for a PTF of 60 G.

(1) Torres, O.; Martin, M.; Sola, E. *Organometallics* **2009**, *28*, 863-870.

(2) Duckett, S. B.; Sleigh, C. J. *Prog. Nucl. Magn. Reson. Spectrosc.* **1999**, *34*, 71-92.

(3) Cowley, M. J.; Adams, R. W.; Atkinson, K. D.; Cockett, M. C. R.; Duckett, S. B.; Green, G. G. R.; Lohman, J. A. B.; Kerssebaum, R.; Kilgour, D.; Mewis, R. E. *JACS* **2011**, *133*, 6134–6137.

(4) Adams, R. W.; Duckett, S. B.; Green, R. A.; Williamson, D. C.; Green, G. G. R. *J. Chem. Phys.* **2009**, *131*, 194505.
